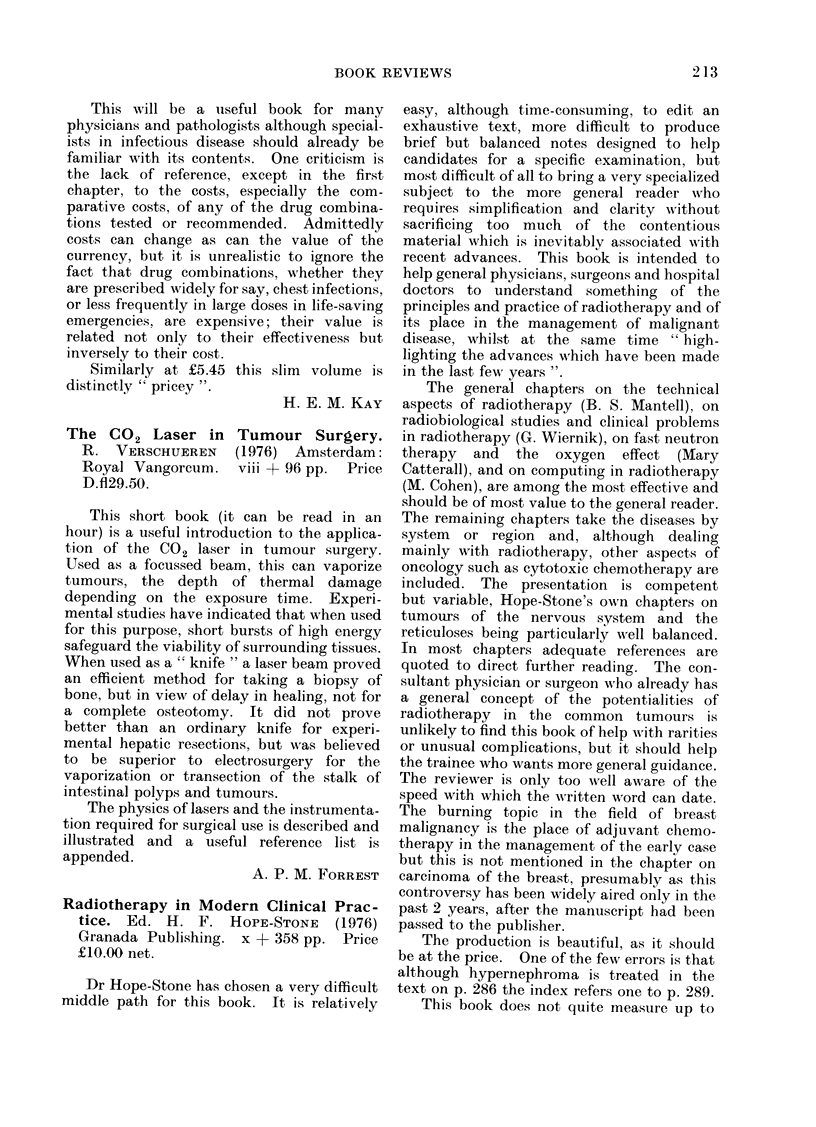# The CO_2_ Laser in Tumour Surgery

**Published:** 1976-08

**Authors:** A. P. M. Forrest


**The $CO_2$ Laser in Tumour Surgery.** R. VERSCHUEREN (1976) Amsterdam: Royal Vangorcum. viii + 96 pp. Price D.fl29.50.

This short book (it can be read in an hour) is a useful introduction to the application of the $CO_2$ laser in tumour surgery. Used as a focussed beam, this can vaporize tumours, the depth of thermal damage depending on the exposure time. Experimental studies have indicated that when used for this purpose, short bursts of high energy safeguard the viability of surrounding tissues. When used as a " knife " a laser beam proved an efficient method for taking a biopsy of bone, but in view of delay in healing, not for a complete osteotomy. It did not prove better than an ordinary knife for experimental hepatic resections, but was believed to be superior to electrosurgery for the vaporization or transection of the stalk of intestinal polyps and tumours.

The physics of lasers and the instrumentation required for surgical use is described and illustrated and a useful reference list is appended.

A. P. M. FORREST